# High genome diversity of *Klebsiella pneumoniae* strains isolated from a Chinese traditional medicine hospital in Jiangsu province, China, from 2023 to 2024

**DOI:** 10.3389/fmicb.2025.1575216

**Published:** 2025-07-09

**Authors:** Yitong Wang, Si Qin, Hongxiong Guo

**Affiliations:** ^1^School of Public Health, Nanjing Medical University, Nanjing, China; ^2^Nanjing Lishui District Hospital of Traditional Chinese Medicine, Nanjing, China; ^3^Jiangsu Provincial Center for Disease Control and Prevention, Nanjing, China

**Keywords:** *Klebsiella pneumoniae*, virulence genes, drug resistance phenotype, hyperviscosity phenotype, antimicrobial

## Abstract

**Objective:**

*Klebsiella pneumoniae* (KP) poses a global health threat, with variations observed across different regions. However, data on the genomic characteristics and drug resistance profiles of KP isolates in Eastern China are limited. To address this gap, we collected KP isolates from a traditional Chinese medicine hospital in Jiangsu Province, China, in order to characterize these features.

**Methods:**

From February 2023 to February 2024, 117 KP isolates were collected from a Chinese traditional medicine hospital in Jiangsu province, China. Antimicrobial susceptibility testing and whole-genome sequencing were performed on all isolates. Then, capsular serotype, multi-locus sequence typing, virulence genes, and resistance genes were identified using Prokka and Kleborate software tools. The hypermucoviscosity phenotype was detected using the string test. Antimicrobial susceptibility was tested following the guidelines by the Clinical and Laboratory Standards Institute, using a commercially prepared and dehydrated panel. A chi-squared test was performed to assess the differences in virulence gene profiles between hypermucoviscous and non-hypermucoviscous isolates.

**Results:**

A high resistance rate to ampicillin (98.7%) and doxycycline (41.0%) was observed in the KP strains. The resistance rate to cephalosporins ranged from 10 to 20%, and 23.7% of the strains were multidrug-resistant (MDR). A total of 66 resistance genes from 13 categories were identified, including carbapenem-resistant genes in four KP strains. The most common capsular serotypes were K1 and K2 (24.0% of the isolates). O1 was the dominant O antigen type (52.1%). The KP strains were classified into 62 sequence types (STs), with ST23, ST29, ST412, and ST111 being the most prevalent (each accounting for >5.0% of the isolates). The carriage rates of the virulence genes aerobactin, yersiniabactin, colibactin, salmochelin, RmpADC, and RmpA2 were 44.4, 47.9, 11.1, 54.7, 55.6, and 31.6%, respectively. All ST23 KP strains carried virulence plasmids other than RmpADC. Hypermucoviscous (HM) KP was observed in 23.4% of the isolates. HM KP strains carried a higher number of virulence genes compared to the non-HM KP strains.

**Conclusion:**

Although no single dominant (KP) clone, serotype, or sequence type was identified among the isolated KP strains from this hospital, the overall resistance rates remained relatively low. However, the relatively prevalent ST23 KP strains, which carry multiple virulence genes, are a concern due to their potential to acquire carbapenem-resistant genes.

## 1 Introduction

*Klebsiella pneumoniae* (KP), a species of the *Klebsiella* genus in the family Enterobacteriaceae, is one of the most important pathogens causing community- and hospital-acquired infections (Wang et al., 2020). The increasing prevalence of carbapenem-resistant strains, particularly among hypervirulent variants, poses a serious challenge to conventional treatment methods (Zhang et al., 2018). The World Health Organization (WHO) has identified carbapenem- and cephalosporin-resistant KP as a critical priority pathogen.^1^ In China, carbapenem-resistant KP (CRKP) accounts for 70–90% of carbapenem-resistant Enterobacteriaceae isolates in some regions (Ouyang et al., 2019; Hu and Wang, 2022; Chen et al., 2024). Furthermore, 40–50% of deaths among immunocompromised patients are linked to carbapenem-resistant KP (Shen et al., 2023; Maierhaba et al., 2025).

KP commonly develops drug resistance by acquiring carbapenemase genes from other bacteria. This genetic exchange also includes genes related to membrane penetration, making KP more difficult to treat. Resistance genes are more commonly found in well-established clonal groups, such as clonal group 258(CG258), which is the most common lineage of carbapenem-resistant KP. ST258 and ST11 are the most frequently observed sequence types (STs) within CG258 KP (Chen et al., 2024; Biakej-Davenet et al., 2014). Similarly, the highly virulent phenotype of KP is often associated with specific K antigen types and the presence of virulence genes (Wyres et al., 2020). The distribution of both resistance genes and virulence genes exhibits significant geographical variation (Tang et al., 2020; Surgers et al., 2016).

Despite being an economically developed region in Eastern China with over 80 million inhabitants, there are limited data on the genomic characteristics and drug resistance profiles of KP isolates in Jiangsu Province. This study analyzed the genome sequences of 117 KP strains isolated over a 1-year period to identify their molecular and phenotypic properties.

## 2 Materials and methods

### 2.1 Bacterial strain isolation

Clinical specimens were collected randomly between February 2023 and February 2024, inoculated onto blood plates, and cultured at 37°C for 24 h. KP strains were identified using the BACTEC 9120 automated bacterial culture system (Becton Dickinson Diagnostic Instrument Systems, Sparks, MD), following the manufacturer’s instructions.

### 2.2 Strain identification and antimicrobial susceptibility

Single colonies of KP were picked with a sterile loop and resuspended in 0.9% NaCl. The bacterial suspension was prepared to achieve a turbidity of 0.5–0.6 McFarland standards. Species identification and antimicrobial susceptibility testing were performed using the VITEK 2 Compact System and the associated reagents from bioMérieux (France), following the manufacturer’s instructions (The Clinical and Laboratory Standards Institute Subcommittee on Antimicrobial Susceptibility Testing, 2020).

### 2.3 Whole-genome sequencing

The strains identified as KP were sent to Sangyo Bioengineering (Shanghai) Co. Whole-genome sequencing was performed using the Illumina HiSeq™ platform. Raw data were quality-controlled using fastp (v0.23.4) and then spliced using SPAdes (3.5.0) to obtain the bacterial genome frame map. The assembled genome sequences were predicted and annotated using Prokka (1.4.15) for gene prediction; the distribution of resistance genes in the strains was predicted using Kleborate (v2.4.1), which is a KP typing tool. Default settings were applied for all the software tools mentioned above.

### 2.4 String test

An inoculating loop was dipped into fresh *Klebsiella pneumoniae* colonies grown on blood agar plates, and the loop was then lifted upward. If the colony formed mucus filaments ≥5 mm in length, the string test was considered positive and the strain was classified as hypermucoviscous *Klebsiella pneumoniae* (hmKP). Conversely, if the filaments were shorter than 5 mm, the test was considered negative and the strain was classified as non-hypermucoviscous *Klebsiella pneumoniae* (non-hmKP).

### 2.5 Statistical methods

Differences in virulence gene carriage and resistance scores between strains with a high-mucus phenotype and those with non-high-mucus phenotype were analyzed using a chi-squared test or Fisher’s exact test. Differences were considered statistically significant at a *p* ≤ 0.05.

## 3 Results

### 3.1 Basic characteristics of patients with KP infection

Key demographic and clinical characteristics were analyzed for a group of 117 patients. Male individuals constituted a significant majority (66.7%, 78 patients), and the majority of the patients (92.3%) were older than 50 years. The largest age group was those over 80 years (32.5%), followed by the 51–70 (30.8%) and 71–80 (29.1%) age groups. Nervous system diseases were the most common initial diagnosis (25.6%), followed by respiratory tract infections (21.4%) and tumors (13.7%). Notably, intracranial hemorrhage (11.1% of total initial diagnoses) and cerebral infarction (6.8%) were the most common nervous system disorders. Lower respiratory tract infections were the predominant type of respiratory illness (accounting for 18.8% of total initial diagnoses). The sites of bacterial isolation most frequently included the respiratory tract (68.4%), followed by the urinary tract (16.2%) and the bloodstream (10.3%) (Table 1).

**Table 1:** Basic characteristics of patients and the sites of bacterial isolation.

Variables	*n*	%
Sex		
Male	78	66.7
Female	39	33.3
Age		
≤50	9	7.7
51–70	36	30.8
71–80	34	29.1
≥81	38	32.5
Primary diagnosis		
Tumor	16	13.7
Nervous system diseases		25.6
Intracranial hemorrhage	13	11.1
Cerebral infarction	8	6.8
Others	9	7.7
Respiratory tract infection		21.4
LRTI*	22	18.8
URTI*	3	2.6
Non-infectious respiratory diseases	8	6.8
Cardiovascular diseases	6	5.1
Liver and/or biliary disease	5	4.3
Diabetes	4	3.4
Others	23	19.7
Bacterial isolation site		100
Respiratory tract	80	68.4
Urinary tract	19	16.2
Blood	12	10.3
Vagina	2	1.7
Perianal area	2	1.7
Liver	1	0.9
Abdominal cavity	1	0.9

LRTI*: Lower respiratory tract infection; URTI*: Upper respiratory tract.

### 3.2 Capsule type

The 117 strains belonged to 45 K capsule types, with K1 and K2 being the most prevalent, each accounting for 12% of the strains. They were followed by K54 and K57, each accounting for 7.7%, and K63, accounting for 5.1% of the strains. The remaining 49.6% (58/117) of the strains were categorized into 38 K capsule types, with each type accounting for 0.85–1.70% of the isolates. O antigens were clearly typed in 88.0% (103/117), with O1 being the most dominant, accounting for 52.1%. This was followed by O3 and O2 types, accounting for 17.9 and 14.5%, respectively. O4 and O5 antigen types were the least common, each accounting for 1.7% of the strains (Supplementary material 1).

### 3.3 Multilocus sequence typing (MLST)

The 117 strains were classified into 62 STs. The most common STs included ST23 (7.7%, 9/117), ST29 (6.0%, 7/117), ST412 (6.0%, 7/117), ST111 (5.1%, 6/117), ST65 (4.3%, 5/117), and ST37 (3.4%, 4/117). The strains ST15, ST17, and ST45 each accounted for 2.6% (3/117) of the isolates. All other STs were identified in 1–2 isolates. No single dominant strain was identified (Supplementary material 2).

All nine ST23 strains were identified as serotype O1: K1 and had the highest virulence gene score. In addition, two of the four ST65 strains also had the highest virulence gene score, with all of their serotypes classified as O1: K2.

### 3.4 Drug resistance phenotype

The 117 KP strains exhibited high resistance rates to ampicillin and doxycycline, at 98.7 and 41.0%, respectively. Resistance rates to minocycline, ciprofloxacin, cotrimoxazole, and ampicillin/sulbactam ranged from 20 to 40%. Resistance rates of KP strains to cefazolin, ceftriaxone, levofloxacin, amitraz, ticarcillin/clavulanic acid, and cefoperazone/sulbactam ranged from 10 to 20%. The remaining rates of antimicrobial resistance were below 10%. A total of 28 out of the 117 KP strains were multidrug-resistant (MDR), with a detection rate of 23.7%. In addition, 15 strains were carbapenem-resistant KP (CRKP), with a detection rate of 12.7% (see Table 2).

**Table 2:** Results of antimicrobial susceptibility testing of the 117 *Klebsiella pneumoniae* strains.

Antibacterial drug name	Sensitive strains	Intermediate strains	Resistant strains	Resistance rate (%)
Ticarcillin/Clavulanic Acid	32	3	4	10.3 (4/39)
Ampicillin	1	0	78	98.7 (78/79)
Ampicillin/sulbactam	60	2	17	21.5 (17/79)
Piperacillin tazobactam	111	0	7	5.9 (7/117)
Cefazolin	66	0	13	16.5 (13/79)
Ceftazidime	105	2	11	9.4 (11/117)
Ceftriaxone	66	0	13	16.5 (13/79)
Cefotetan	78	0	1	1.3 (1/79)
Cefoperazone/sulbactam	34	1	4	10.3 (4/39)
Cefepime	107	0	11	9.4 (11/117)
Imipenem	103	12	3	2.5 (3/117)
Meropenem	37	0	2	5.1 (2/39)
Ertapenem	78	0	1	1.3 (1/79)
Aztreonam	102	1	14	12.0 (14/117)
Levofloxacin	79	22	16	13.7 (16/117)
Ciprofloxacin	82	5	31	26.5 (31/117)
Amikacin	112	0	3	2.6 (3/115)
Gentamicin	69	3	7	8.9 (7/79)
Tobramycin	103	6	9	7.7 (9/117)
Cotrimoxazole	92	0	26	22.0 (26/117)
Doxycycline	23	0	16	41.0 (16/39)
Minocycline	19	9	11	28.2 (11/39)
Colistin	38	0	1	2.6 (1/39)
Tigecycline	32	0	1	3.0 (1/33)

### 3.5 Drug resistance gene

**Figure 1:**
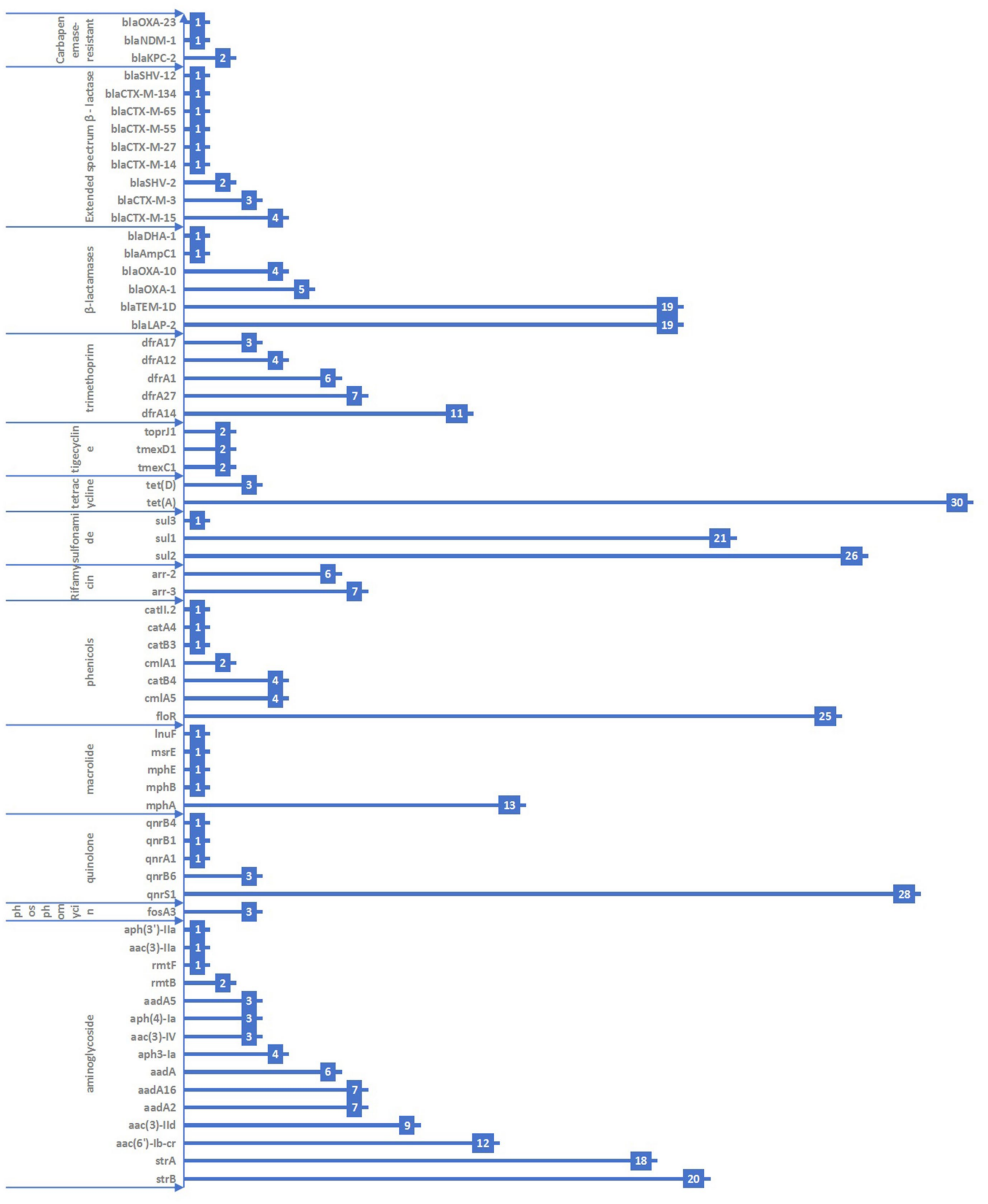
Distribution of antimicrobial resistance genes in the 117 KP strains collected from Jiangsu province, China, 2023–2024.

A total of 66 antimicrobial resistance genes across 13 categories were detected in the 117 KP strains. The most commonly detected genes were *tet*(A)*, qnrS*1*, sul*2, and *floR,* with carriage rates of 25.4, 23.7, 22.0, and 21.2%, respectively. The genes *strB, strA, aac*(6′)*-Ib-cr, mphA, sul1, bla*LAP-2, and *bla*TEM-1D had carriage rates ranging from 10 to 20%. The remaining genes had carriage rates of <10%. The specific types of resistance genes are shown in Figure 1.

A total of 14 KP strains were found to carry extended-spectrum *β*–lactamase (ESBL) resistance genes. These included *bla*CTX-M-15 (four strains, 3.4%), *bla*CTX-M-3 (three strains, 2.5%), *bla*SHV-2 (two strains, 1.7%), *bla*CTX-M-14 (one strain, 0.8%), *bla*CTX-M-27 (one strain, 0.8%), *bla*CTX-M-55 (one strain, 0.8%), *bla*CTX-M-65 (one strain, 0.8%), and *bla*CTX-M-134 (one strain, 0.8%). Furthermore, four strains were found to carry carbapenem-resistant genes, including *bla*KPC-2 (two strains, 1.7%), *bla*NDM-1 (one strain, 0.8%), and blaOXA-23 (one strain, 0.8%).

We also assessed the consistency between phenotypic and genotypic drug resistance. It revealed significant differences (*p* < 0.05) for aminoglycosides, *β*-lactams, sulfonamides, and tetracyclines. In contrast, no statistically significant difference (*p* > 0.05) was observed for carbapenems and quinolones. The consistency for carbapenems and quinolones was moderate, with Kappa values of 0.559 and 0.597, respectively (as shown in Table 3).

**Table 3:** Consistency test of phenotypic and genotypic resistance.

Types of antimicrobial drugs	Phenotypic resistance		Genotypic resistance		*Kappa* value	*P-*value
	Number	Drug resistance rate (%)	Number	Carrier rate (%)		
Aminoglycosides	13	11.0	33	27.3	0.380	<0.001*
*β*-lactams	84	71.2	38	32.0	−0.033	<0.001*
Carbapenems	3	2.5	4	3.4	0.559	1.000
Quinolones	31	26.3	34	28.8	0.597	0.648
Sulfonamides	26	22.0	37	31.4	0.636	0.013*
Tetracyclines	16	13.6	33	28.0	0.276	0.002*

^*^Indicates statistically significant differences.

### 3.6 Virulence gene

**Figure 2:**
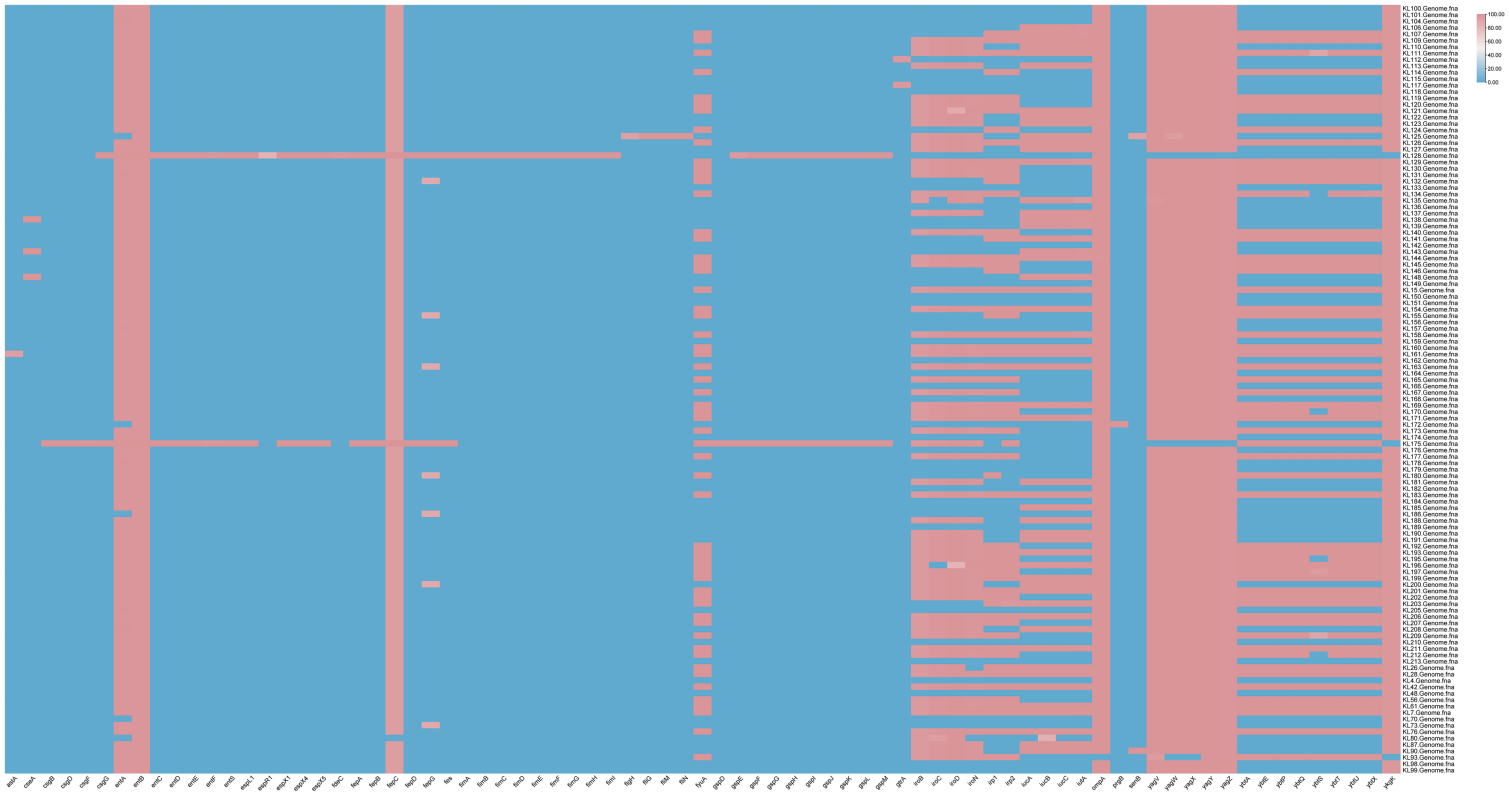
Virulence gene profiles of the 117 KP strains collected from Jiangsu province, China, between 2023 and 2024.

Of the 117 bacterial strains, 52 carried *aerobactin* plasmids, mainly *iuc*1 (37 strains) and *iuc*3 (15 strains). In addition, 56 strains carried *yersiniabactin* plasmids, including *ybt*10, *ybt*14, *ybt*16, *ybt*1, *ybt*22, *ybt*2, *ybt*4, *ybt*5, *ybt*9, and *ybt*17. *ybt4* (21), *ybt1* (10), and *ybt*2 (10) were the major *yersiniabactin* plasmids, with 37.5% (21/56), 17.9% (10/56), and 17.9% (10/56), respectively. A total of 13 bacterial strains carried *colibactin* plasmids, including *clb*2 (76.9%, 10/13) and *clb*3 (23.1%, 3/13). A total of 64 bacterial strains carried *salmochelin* plasmids, mainly *iro*1 (85.9%, 55/64) and *iro*3 (14.1%, 9/64). Furthermore, 65 strains carried the *RmpADC* plasmid (55.6%, 65/117), and 37 strains carried the *RmpA*2 plasmid (31.6%, 37/117) (Supplementary material 3). The majority of the detected virulence genes are involved in the uptake of iron and the synthesis of proteins related to iron transport (Figure 2).

### 3.7 High-mucus phenotype

The string test results showed that 33 of the 117 bacterial strains tested positive, accounting for 28.2%. There were significantly more strains carrying the virulence plasmid in the high-mucus phenotype group than in the non-high-mucus phenotype group (see Table 4). The high-mucus phenotype strains had lower resistance scores compared to the non-high-mucus phenotype strains. Although K1 and K2 serotypes were more prevalent in the high-mucus phenotype strains than in the non-high-mucus phenotype strains, there was no statistically significant difference (*p* > 0.05).

**Table 4:** Correlation analysis between virulence factors and the high-mucus phenotype.

Variable		High-mucus phenotype		Chi-squared	*P*-value
		Yes	No		
Aerobactin	Yes	22	11	7.982	0.005
	No	30	54		
Yersiniabactin	Yes	22	11	5.505	0.019
	No	34	50		
Salmochelin	Yes	31	2	26.397	*p* < 0.001
	No	33	51		
*RmpA*	Yes	31	2	25.304	*P* < 0.001
	No	34	50		
*RmpA2*	Yes	18	15	9.741	*p* = 0.002
	No	19	65		

## 4 Discussion

In this study, we report for the first time the main molecular characteristics and antimicrobial resistance profiles of prevalent KP strains in Jiangsu province, China. Our findings indicate a high diversity of KP isolates, as evidenced by variations in capsule (K and O antigen) serotypes and MLST types. Notably, there was no single serotype or MLST type that predominated among the isolates. While previous research has identified K1 and K2 serotypes as commonly associated with highly virulent KP strains that cause invasive infections (Tian et al., 2022; Lin et al., 2012; Paczosa and Mecsas, 2016), and has explored their capsules as potential virulence factors, this study found no significant difference in the proportion of these two serotypes between hmKP and non-hmKP strains. This lack of difference may be attributed to the broad range of serotypes found in our study, with no clear dominant serotype. Although K1 and K2 were the two most frequently observed serotypes, they collectively represented only 24.0% of all KP strains.

Consistent with the serotype distribution, the KP isolates in this study exhibited a high degree of sequence type diversity, with 62 distinct STs identified and no single ST being dominant. The most frequently identified STs, each representing more than 5% of all isolates, were ST23, ST412, ST29, and ST111. Although ST 23 has been reported as the dominant ST among highly virulent KP isolates in China, often carrying carbapenem-resistant genes (Liu et al., 2023; Wang et al., 2024; Liu et al., 2014), and hypervirulent carbapenem-resistant KP strains pose a significant global public health threat (Yang et al., 2022), no ST23 strains carrying carbapenem-resistant genes were found in this study. This absence highlights the substantial geographic variation in the molecular characterization of KPs.

The resistance rate of KP isolates in this study was below 20% for the majority of the antibiotics tested. Exceptions included ampicillin, doxycycline, minocycline, ciprofloxacin, cotrimoxazole, and ampicillin/sulbactam, all of which exhibited resistance rates above 20%. Imipenem and meropenem showed low resistance rates of 2.5 and 5.1%, respectively, which are lower than those reported in other hospitals nationwide in 2023 (Lei et al., 2024; China Antimicrobial Resistance Surveillance System, 2024). Both ESBL-producing and carbapenem-resistant KP strains also demonstrated low resistance rates in this hospital (Tesfa et al., 2022). The hospital’s recent implementation of strict file management protocols for restricted-use antimicrobials, such as imipenem and meropenem, highlights the importance of standardized antibiotic use in controlling the spread of drug-resistant strains. Interestingly, one CRKP strain carrying the *bla*OXA-23 resistance gene did not exhibit phenotypic resistance, potentially due to the overexpression of *β*-lactamase (Avci et al., 2023). We also observed a discrepancy in the strains whereby the carriage rate of resistance genes was higher than the rate of phenotypic resistance. However, for β-lactam antibiotics, the carriage rate of theβ-lactamase genes was lower than the observed phenotypic resistance. This highlights the complex relationship between a bacterium’s genetic potential for antibiotic resistance and its actual observable resistance in laboratory tests.

The virulence of KP is strongly associated with its virulence gene profile. In this study, the prevalence of several key virulence factors was evaluated: yersiniabactin (44.4%), aerobactin (47.9%), salmochelin (11.1%), colibactin (53.0%), RmpADC (55.6%), and *RmpA2* (31.6%). The majority of the isolates carried one or two virulence genes. As expected, the hvKP strains exhibited a significantly higher carriage rate of all tested virulence genes compared to the cKP strains. Notably, all ST23-type isolates in this study carried five virulence genes, consistent with previous reports that identified ST23 as the dominant sequence type among hvKP strains in China (Zhu et al., 2020; Wang et al., 2022). However, in contrast to previous studies, all ST23-type strains isolated in this study remained susceptible to all tested antibiotics except ampicillin.

## 5 Conclusion

KP endemic to this region exhibits significant diversity in both serotypes and MLST, with no single strain being dominant. While virulence gene carriage was generally high, individual virulence genes displayed multiple allelic genotypes. K1-ST23 KP, characterized by high virulence, was the most frequently detected strain, raising concerns about their potential to evolve into highly virulent strains that could be resistant to both carbapenems and ESBL antibiotics. Furthermore, given the high resistance of KP to ampicillin and doxycycline, it is crucial to address this concern and implement necessary prevention and control strategies.

## Data Availability

The datasets used in this study are available from the corresponding author on reasonable request.

## Footnotes

^1^ https://iris.who.int/bitstream/handle/10665/376776/9789240093461-eng.pdf
