# Supplementary material for: High genome diversity of Klebsiella pneumoniae strains isolated from a Chinese traditional medicine hospital in Jiangsu province, China, from 2023 to 2024
Source: Front Microbiol. 2025 Jul 9;16:1575216. doi: 10.3389/fmicb.2025.1575216 (PMC12283684; doi:10.3389/fmicb.2025.1575216)
Supplement: Supplementary file 1 [file Table_1.docx]

Supplement 1

Distribution of different types of K and O antigens of 117 *KP* strains

| K type | n | K type | n | K type | n | K type | n | O type | n |
| --- | --- | --- | --- | --- | --- | --- | --- | --- | --- |
| K1 | 14 | K124 | 2 | K120 | 1 | K8 | 1 | O1 | 63 |
| K2 | 14 | K52 | 2 | K123 | 1 | K103 | 1 | O3 | 21 |
| K54 | 9 | K7 | 2 | K125 | 1 | K107 | 1 | O2a | 17 |
| K57 | 9 | K102 | 2 | K127 | 1 | K110 | 1 | O101 | 7 |
| K63 | 6 | K5 | 2 | K142 | 1 | K114 | 1 | O103 | 3 |
| K24 | 4 | K10 | 1 | K31 | 1 | K165 | 1 | O12 | 2 |
| K62 | 4 | K11 | 1 | K35 | 1 | K183 | 1 | O104 | 2 |
| K48 | 3 | K12 | 1 | K38 | 1 | K3 | 1 | O4 | 1 |
| K64 | 3 | K14 | 1 | K39 | 1 | K30 | 1 | O5 | 1 |
| K20 | 2 | K15 | 1 | K43 | 1 | K71 | 1 |  |  |
| K21 | 2 | K16 | 1 | K49 | 1 | Unknown | 2 |  |  |
| K23 | 2 | K17 | 1 | K55 | 1 |  |  |  |  |
| K25 | 2 | K18 | 1 | K61 | 1 |  |  |  |  |
