# Supplementary material for: High genome diversity of Klebsiella pneumoniae strains isolated from a Chinese traditional medicine hospital in Jiangsu province, China, from 2023 to 2024
Source: Front Microbiol. 2025 Jul 9;16:1575216. doi: 10.3389/fmicb.2025.1575216 (PMC12283684; doi:10.3389/fmicb.2025.1575216)
Supplement: Supplementary file 2 [file Table_2.docx]

Supplement 2

Multilocus sequence typing of KP isolates

| Sequence type | n | Sequence type | n | Sequence type | n | Sequence type | n |
| --- | --- | --- | --- | --- | --- | --- | --- |
| ST23 | 9 | ST700 | 2 | ST464 | 1 | ST1229 | 1 |
| ST412 | 8 | ST86 | 2 | ST469 | 1 | ST1265 | 1 |
| ST29 | 7 | ST611 | 2 | ST484 | 1 | ST1266 | 1 |
| ST111 | 6 | ST43 | 1 | ST519 | 1 | ST1569 | 1 |
| ST65 | 5 | ST55 | 1 | ST592 | 1 | ST1843 | 1 |
| ST37 | 4 | ST101 | 1 | ST626 | 1 | ST1948 | 1 |
| ST15 | 3 | ST147 | 1 | ST639 | 1 | ST2665 | 1 |
| ST17 | 3 | ST160 | 1 | ST660 | 1 | ST2729 | 1 |
| ST45 | 3 | ST194 | 1 | ST716 | 1 | ST3890 | 1 |
| ST1049 | 2 | ST202 | 1 | ST721 | 1 | ST3132 | 1 |
| ST1056 | 2 | ST299 | 1 | ST726 | 1 | ST3687 | 1 |
| ST11 | 2 | ST307 | 1 | ST727 | 1 | ST4552 | 1 |
| ST1107 | 2 | ST314 | 1 | ST873 | 1 | ST5022 | 1 |
| ST20 | 2 | ST327 | 1 | ST906 | 1 | ST5391 | 1 |
| ST25 | 2 | ST367 | 1 | ST950 | 1 | ST5571 | 1 |
| ST375 | 2 | ST414 | 1 | ST1023 | 1 | ST7099 | 1 |
| ST540 | 2 | ST420 | 1 | ST1184 | 1 |  |  |

Note: underline indicates the sequence type more than 3 isolates
