# Supplementary material for: High genome diversity of Klebsiella pneumoniae strains isolated from a Chinese traditional medicine hospital in Jiangsu province, China, from 2023 to 2024
Source: Front Microbiol. 2025 Jul 9;16:1575216. doi: 10.3389/fmicb.2025.1575216 (PMC12283684; doi:10.3389/fmicb.2025.1575216)
Supplement: Supplementary file 3 [file Table_3.docx]

Supplement 3

Carriage rate of virulence genes of 117 KP strains

| Virulence genes | | n | % |
| --- | --- | --- | --- |
| Yersiniabactin | total | 61 | 52.1 |
|  | *ybt4* | 21 |  |
|  | *ybt1* | 10 |  |
|  | *ybt2* | 10 |  |
|  | other | 20 |  |
| Colibactin | total | 13 | 11.1 |
|  | *clb2* | 10 |  |
|  | *clb3* | 3 |  |
| Salmochelin | **total** | 64 | 54.7 |
|  | *iro1* | 54 |  |
|  | *iro3* | 9 |  |
|  | unkonwn | 1 |  |
| Aerobactin | **total** | 52 | 44.4 |
|  | *iuc1* | 37 |  |
|  | *iuc3* | 15 |  |
| *RmpADC* | | 65 | 55.6 |
| *RmpA2* | | 37 | 31.6 |
